# Enhanced UV photosensitivity from rapid thermal annealed vertically aligned ZnO nanowires

**DOI:** 10.1186/1556-276X-6-504

**Published:** 2011-08-22

**Authors:** Soumen Dhara, PK Giri

**Affiliations:** 1 Department of Physics, Indian Institute of Technology, Guwahati, Guwahati-781039, India; 2 Centre for Nanotechnology, Indian Institute of Technology, Guwahati, Guwahati-781039, India

**Keywords:** ZnO Nanowires, rapid thermal annealing, photocurrent, photoresponse

## Abstract

We report on the major improvement in UV photosensitivity and faster photoresponse from vertically aligned ZnO nanowires (NWs) by means of rapid thermal annealing (RTA). The ZnO NWs were grown by vapor-liquid-solid method and subsequently RTA treated at 700°C and 800°C for 120 s. The UV photosensitivity (photo-to-dark current ratio) is 4.5 × 10^3 ^for the as-grown NWs and after RTA treatment it is enhanced by a factor of five. The photocurrent (PC) spectra of the as-grown and RTA-treated NWs show a strong peak in the UV region and two other relatively weak peaks in the visible region. The photoresponse measurement shows a bi-exponential growth and bi-exponential decay of the PC from as-grown as well as RTA-treated ZnO NWs. The growth and decay time constants are reduced after the RTA treatment indicating a faster photoresponse. The dark current-voltage characteristics clearly show the presence of surface defects-related trap centers on the as-grown ZnO NWs and after RTA treatment it is significantly reduced. The RTA processing diminishes the surface defect-related trap centers and modifies the surface of the ZnO NWs, resulting in enhanced PC and faster photoresponse. These results demonstrated the effectiveness of RTA processing for achieving improved photosensitivity of ZnO NWs.

## Introduction

ZnO nanostructures (NS) such as nanowires (NWs), nanorods, thinfilm, etc. are extensively studied for their applications in various optoelectronic devices, e.g., UV photodetectors, UV light emitting diodes, phototransistors etc. [1-6]. However, photodetection and photoconductivity of the ZnO NWs depends on the surface condition, structural quality, and the growth methods. For the as-grown ZnO NS, the UV photosensitivity and photoresponse are below the required level for real-time device application [7-9]. Efforts are being made to improve the UV photosensitivity and photoresponse of the ZnO NS. It is known that the photoconduction in the ZnO NS is controlled by oxygen adsorption and desorption on the surface of the ZnO NS [4,10,11]. Therefore, surface modification or structural improvement can improve the photosensitivity as well as photoresponse of ZnO NWs. Various groups have put efforts to enhance the photoresponse and photosensitivity by using appropriate dopant [12], surface passivation using ZnS coating [13], polyacrylonitrile/L-lysine treatment [1,14], integrating ultrathin metal nanoparticles layer [5,15], and making ZnO nanorod/graphene heterostructure [16]. Since the as-grown ZnO NS contains surface defects which are basically trap centers for carriers, during UV excitation, the trap centers easily trap the photocarriers resulting in low photocurrent (PC) as well as weak photoluminescence (PL). In a recent study, we found that defect-related trap centers on the ZnO NWs surface can be considerably removed by employing RTA processing and an enhanced band-edge PL could be obtained [17]. As a consequence, it is expected that RTA-treated ZnO NWs can show enhanced UV photosensitivity. Here, we report on the effect of RTA on the enhanced photoconduction and photoresponse behavior of the vertically aligned ZnO NWs and the origin of the enhanced PC is explained based on the experimental results. The bi-exponential growth and decay time constants of the PC are carefully analyzed and the mechanism of the observed faster growth and decay after RTA are explained through a suitable model.

## Experimental

ZnO NWs were synthesized by a vapor-liquid-solid method using gold catalyst. Details of the experimental process and growth parameters are discussed elsewhere [18]. In brief, commercial ZnO nanopowder (Sigma-Aldrich, USA, purity 99.999%, average size 50-70 nm) was used as source material and vapor deposition was carried out in a horizontal muffle furnace. The ZnO vapor was formed at 950°C and was deposited on the ultrathin gold coated (approximately 2 nm thick) *n-*type Si (100) substrate, which was placed downstream at 750°C. The Si(100) substrate was pre-cleaned by standard method followed by HF etching to remove the native oxide layer. After the ZnO deposition, morphology and structure of the samples were analyzed using field emission scanning electron microscopy (FESEM, Sigma, Zeiss, Oberkochen, Germany) and transmission electron microscopy (TEM, JEM2100, JEOL, Tokyo, Japan) with selected area electron diffraction (SAED). The SEM and TEM images confirm the vertical growth of ZnO NWs on the Si substrate. Subsequently, the as-grown ZnO NWs was subjected to RTA processing at 700°C and 800°C for 120 s in Ar gas ambient using a commercial RTA system (Mila3000, Ulvac, Yokohama, Japan). During RTA process, the heating and cooling rate was kept at 30°C/s. For the PC measurements, a 25-μm-thick gold wire was used for electrical contact on the top of the ZnO NWs array using good quality silver paste with diameter of the circular contact area approximately 400 μm. The distance between the two electrodes was 1.5 mm. A good Ohmic contact was obtained after annealing at 200°C for 10 min in Ar ambient. For the current measurement on RTA-treated NWs, two new contacts with similar specifications were made near the old contacts on top of the ZnO NWs. The photoresponse was measured using a picoammeter (Model 6487, Keithley, Aurora Road, Cleveland, Ohio) under the illumination of monochromated UV light (wavelength 360 nm) from a 150 W xenon lamp at a light intensity of approximately 0.5 mW/cm^2 ^in ON and OFF conditions. The UV light is tightly focused onto the sample making sure that the region between the two electrodes is only illuminated. The PC spectra were recorded in the excitation range of 300 to 700 nm. The specular reflectance of the as-grown and RTA-treated ZnO NWs was measured using a UV-Vis spectrometer (Varian Carry 50, Varian Inc., Palo Alto, CA, USA). The PL spectra of the as-grown and RTA-treated samples were recorded with a 325 nm He-Cd laser excitation using a high-resolution commercial PL spectrometer (FS 920P, Edinburg Instruments, Kingston, UK). For comparative analysis, PL measurements on all samples were done under identical experimental conditions. All the measurements were carried out at room temperature and atmospheric pressure.

## Results and discussion

**Figure 1:**
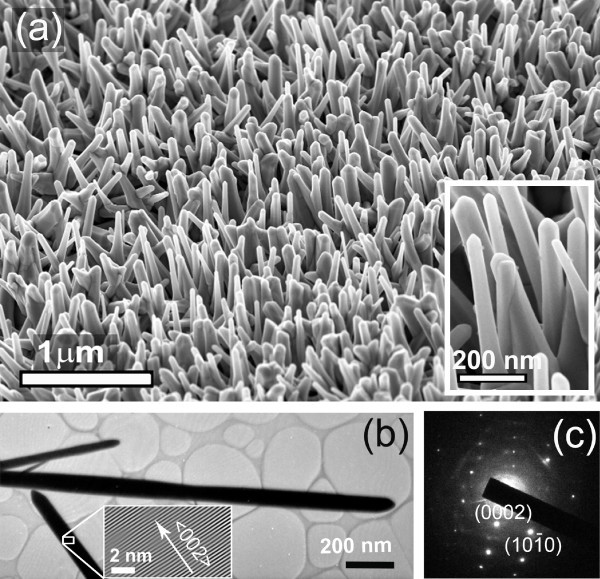
**FESEM and TEM images of the vertically grown ZnO NWs**. **(a) **FESEM image (tilted view) of the as-grown ZnO NWs, that are grown vertically to the Si(100) substrate. Inset shows the NWs at higher magnification. **(b) **and **(c) **show the typical TEM image and SAED pattern of the as-grown ZnO NWs. The inset of (b) shows the high-resolution lattice image of the corresponding NW.

Figure 1a shows the FESEM images of the as-grown ZnO NWs grown at 750°C. The diameter of the NWs is in the range of 50 to 80 nm and length about a few microns. As seen from the images, the NWs grew vertically aligned to the Si(100) substrate plane. Inset shows the ZnO NWs at higher magnification. Note that vertically aligned NWs have several important applications and are preferred for direct integration into the fabrication of real devices over large scale [19]. Figure 1b shows the typical TEM image of the ZnO NWs. The inset shows the high-resolution lattice fringe image of the selected area of the ZnO NWs. Figure 1c shows the SAED pattern of one of the ZnO NW. The SAED pattern (Figure 1c) and the high-resolution lattice image of the NWs (inset of Figure 1b) confirms the crystalline hexagonal structure of the NWs and growth direction is along < 002 >. After the RTA processing, SEM images do not show any significant change in morphology, while the sizes of the ZnO NWs were marginally increased. The structural characterization of the as-grown and RTA-treated ZnO NWs was done by using XRD and Raman spectroscopy techniques. The observed structural changes in the RTA-treated NWs are discussed in our previous study [17]. The XRD studies on the as-grown and RTA-treated NWs shows the c-axis orientation. After the RTA processing, the intensity of the XRD pattern increases and the full width at half maxima (FWHM) of the (002) peak decreases slightly, indicating improved crystallinity and reduced strain of the RTA processed samples. The Raman spectrum of the ZnO NWs shows strong $E_{2}^{\text{high}}$phonon mode along with the other phonon modes of wurtzite ZnO phase. The RTA processing shows a higher intensity of the $E_{2}^{\text{high}}$mode due to improved crystallinity. Note that the Raman spectra of the RTA processed NWs also clearly indicates a reduction in strain.

**Figure 2:**
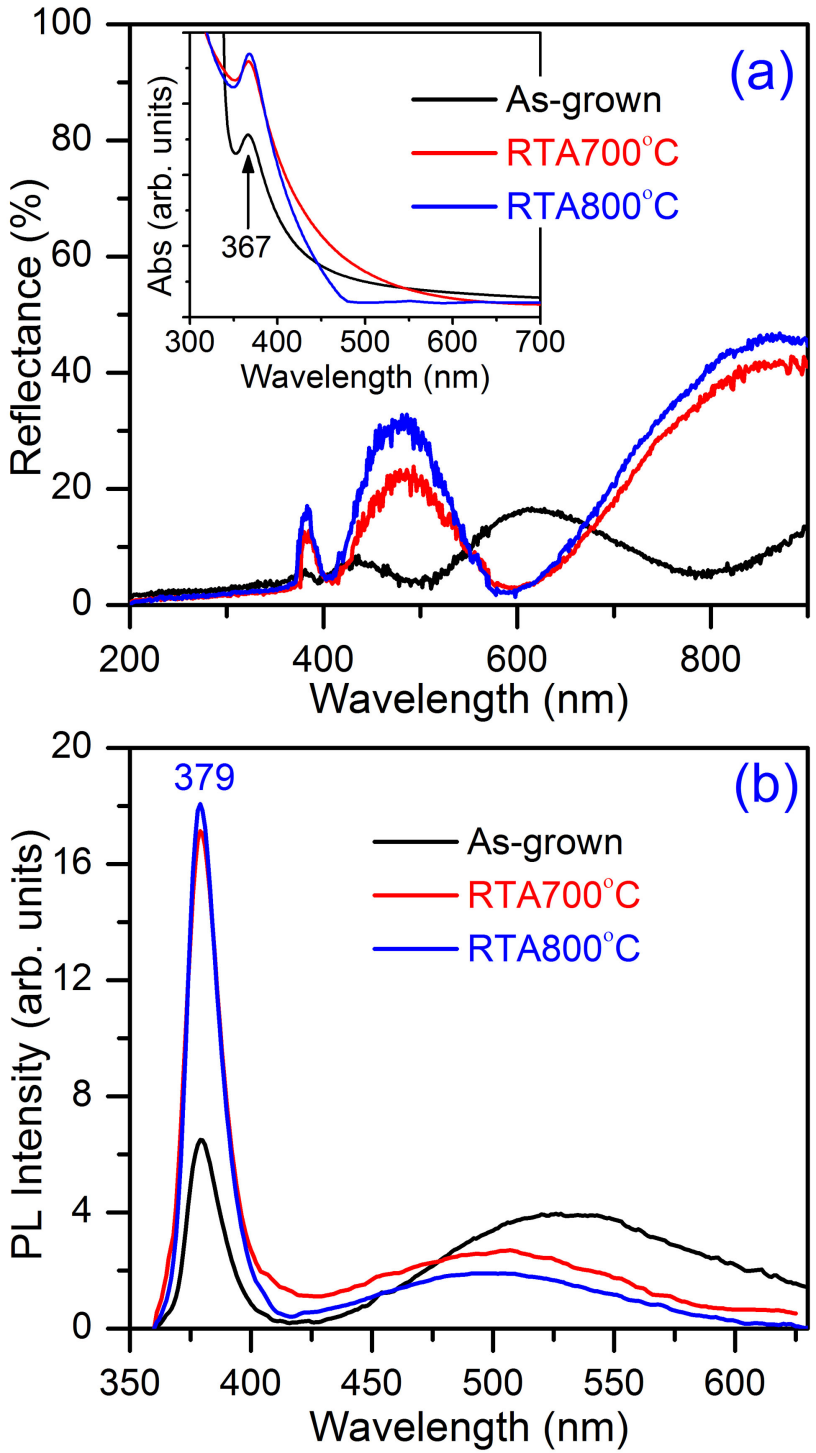
**UV-Vis absorption and photoluminescence spectra of the as-grown and RTA-treated ZnO NWs**. **(a) **UV-Vis specular reflectance spectra of the as-grown and RTA-treated ZnO NWs grown at 750°C. Inset shows the calculated absorbance spectra of the corresponding NWs from reflectance spectra. **(b) **Room temperature PL spectra of the as-grown and RTA-treated ZnO NWs. A significant enhancement in UV emission and decrease in green emission are observed after RTA treatment.

Figure 2a shows the UV-Vis specular reflection spectra of the as-grown and RTA-treated ZnO NWs. It shows a maximum reflectance of approximately 16% in the 200 to 900 nm range for the as-grown NWs, whereas after RTA, it increases to 40%. This increase of the reflectance can be explained on the basis of modification in the structural characteristics (diameter of the NWs, surface roughness, etc) during RTA treatment. Note that after RTA processing, the reflectance magnitude is reduced below the bandgap wavelength of ZnO (approximately 367 nm, discussed later) and enhanced above the bandgap wavelength, which indicates an enhancement in absorption intensity below the bandgap wavelength and a reduction in absorption intensity above this wavelength. This reduced absorption in the visible region clearly indicates the reduction of oxygen vacancy states after RTA processing. Note that reflectance values in the UV region (200 to 400 nm) is quite low (average value approximately 2.7%), which indicates a very high absorption in this region. The high UV-absorption of the synthesized ZnO NWs demonstrates its suitability for the use in UV photodetctors. Huang et al. [20] reported similar reflection spectra of the hydrothermally grown ZnO NWs with highest reflection of 18%. The absorption spectra was calculated from the above specular reflectance spectra according to the method proposed by Rusli et al. [21]. The absorption calculation was done in the region where *n*_ZnO _<*n*_Si _is satisfied (*n*_ZnO _and *n*_Si_: real part of refractive index of ZnO and Si, respectively). The UV-Vis absorption spectra (inset of Figure 2b) of the as-grown and RTA-treated ZnO NWs show a high absorption in UV region with a sharp peak at 367 nm corresponding to the band-to-band absorption. The observed weak absorption component in the region of 400 to 550 nm is due to the absorption by the trap centers present on the surface of the NWs. After RTA treatment, the intensity of the UV-absorption peak is enhanced by a factor of approximately 1.4, compared to the as-grown case. Therefore, one would expect enhanced UV PL as well as photocurrent in the UV region from the RTA-treated ZnO NWs. Note that visible absorption is considerably reduced in 800°C RTA-treated ZnO NWs, implying significant reduction of the defect density as a result of processing.

The room temperature PL spectra of the as-grown and RTA-treated ZnO NWs are shown in Figure 2b. As-grown and RTA-treated ZnO NWs exhibit strong near band-edge (NBE) UV emission at 380 nm and a broad green emission band. Gaussian multipeak fitting shows the existence of two green emission bands, one at 500 nm and another at 545 nm [17]. The observed NBE emission is due to bound excitonic recombination, and the green emission at approximately 500 nm is due to the presence of oxygen vacancy states on the surface of ZnO NWs and second green emission band is due to presence of deep interstitial oxygen states inside the NWs [22]. More details of these results are reported elsewhere [17]. In an earlier study on SnO_2 _NWs, Kar et al. [23] showed that post-rapid thermal annealing improves the crystalline quality of the NWs due to the decrease of oxygen vacancy states. In our case, we also observed similar structural improvement which results in enhanced band-edge emission and reduced vacancy-related emission intensities. The RTA-treated NWs at 700°C show threefold enhancement of the UV emission peak intensity compared to the as-grown case and the intensity of the green emission is considerably reduced. The NWs, that are RTA treated at 800°C show slight enhancement of UV emission peak intensity and significant reduction of green emission intensity. The intensity ratio of UV-to-visible emission is increased from 2.0 to approximately 4.85 for the RTA at 700°C. Whereas, a five times enhancement of this ratio is observed for the NWs treated at 800°C, compared to the as-grown case. Further analysis shown that after RTA, only first green emission band is survives, while the 2nd green emission band is fully removed. However, one new emission peak is observed at 394 nm after RTA, which corresponds to the recombination at band-tail states [22]. Details of the peak fitting and peak parameters can be found in the Additional file 1.

**Figure 3:**
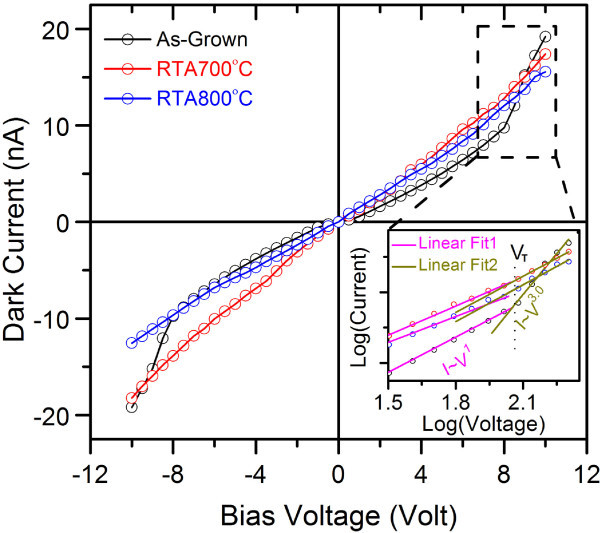
**The dark current-voltage characteristics of as-grown and RTA-treated ZnO NWs processed at 700°C and 800°C**. Inset shows the magnified view of the selected region in log-log scale to highlight the SCLC contribution.

Figure 3a shows the dark current-voltage (I-V) characteristics of the as-grown and RTA-treated ZnO NWs. Inset shows the magnified view of the selected region at logarithmic scale. For the as-grown NWs, it shows an Ohmic behavior (*I *~ *V*^1.0^) up to a bias voltage of ± 8 V with current in the range of nanoampere, beyond which dark current is proportional to*V*^~3.0^. In this case, obtained trap filled limit voltage (*V*_T_) is 8 V. The observed transition in the power dependence of current in the I-V curve is likely to arise from charge carriers trapped in the surface defect states that contribute to the high current at higher bias voltage. The high current at higher bias voltage can be well explained by the space-charge-limited (SCLC) current mechanism. Although, power dependence of 2.0 is considered as SCLC, higher power dependence on voltage can be observed depending on the energy distribution of trap centers [24]. As the PL spectra revealed the presence of trap centers with different energies, the higher power dependence of current on voltage is expected. This behavior is consistent with previous reports on doped metal oxide thin film based resistive switching devices [25,26] and it is explained on the basis of presence of oxygen vacancy-related traps within the bandgap of the materials. Under a negative bias voltage, oxygen vacancies with positive charges migrate away from the interface between contact and the ZnO, which widens the depletion layer, resulting in high resistivity. On the other hand, with positive bias voltage, the oxygen vacancies start moving toward the interface, resulting in higher current. In case of RTA-treated NWs, a nearly linear dark I-V characteristic with slightly high current is observed. Therefore the analysis of the dark I-V characteristics of the ZnO NWs clearly indicates the presence of high density of trap centers on the surface of the as-grown ZnO NWs, while the trap density is significantly reduced in RTA processed ZnO NWs.

**Figure 4:**
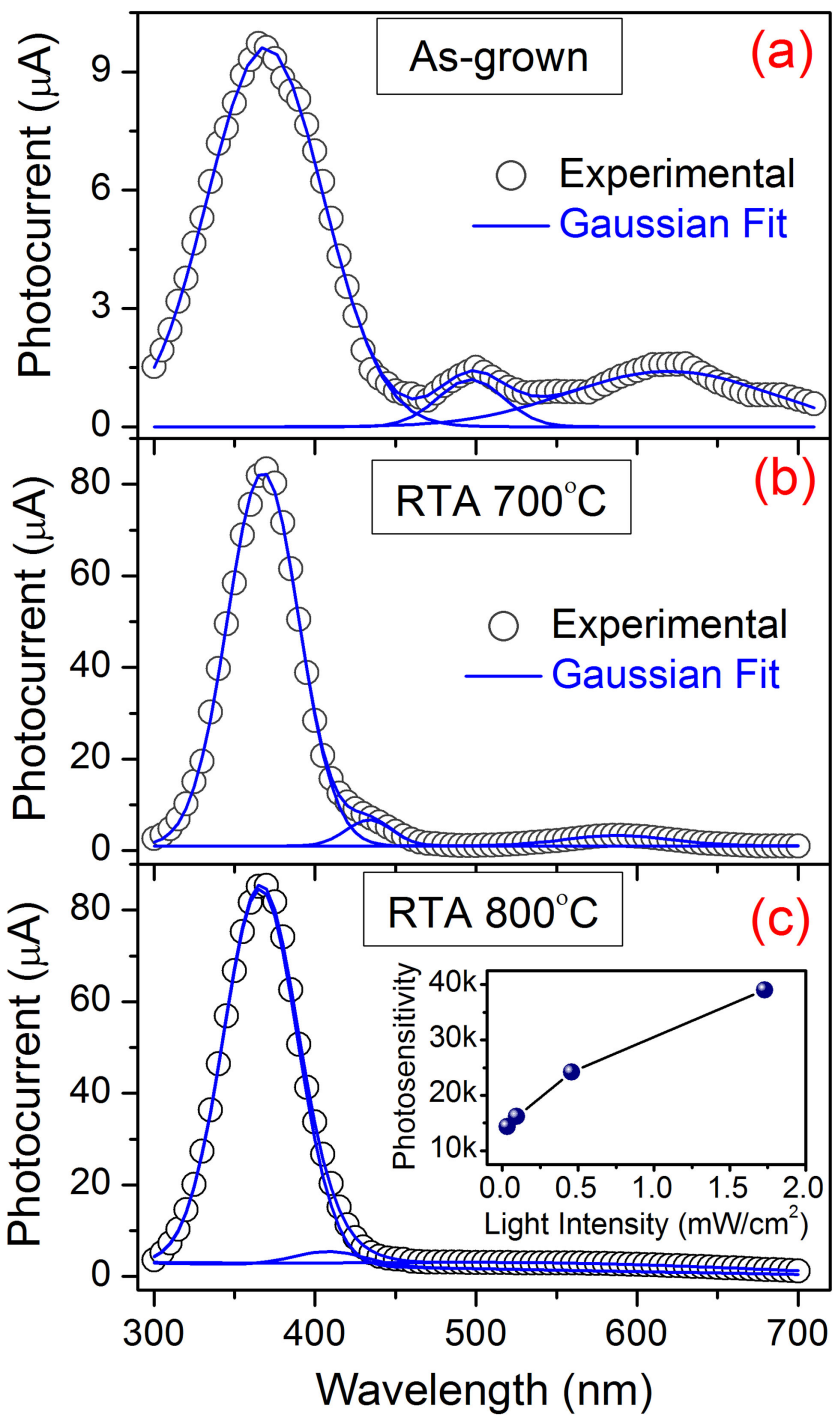
**The photocurrent spectra of the ZnO NWS**. **(a) **as-grown, **(b) **RTA treated at 700°C, and **(c) **RTA treated at 800°C, respectively at a bias voltage of 2.5 V. The inset of (c) shows the variation of photosensitivity at different intensity of UV light (wavelength 369 nm) for the ZnO NWs RTA treated at 800°C.

Figure 4 shows the PC spectra of the as-grown and RTA-treated ZnO NWs, measured at a bias of 2.5 V, which show a strong peak at 369 nm and two other relatively weak peaks in the visible region. The observed strong peak at 369 nm in the PC spectra is due to the band-edge absorption followed by generation of photocarriers (electron-hole pair). The other peaks in the visible region in the PC spectra are fitted with two Gaussian peaks and obtained peak positions are at 496 and 620 nm for as-grown NWs; 433 and 588 nm for RTA-treated NWs at 700°C; and 409 and 561 nm for the RTA NWs at 800°C. The observed peaks in the visible region are due to the generation of carriers from the defect states and band-tail states. The observed PC spectra are consistence with the PL spectra of the as-grown and RTA-treated NWs, which show similar spectra with strong peak in the UV region and relatively weak peak in the visible region. For the as-grown NWs, the UV photosensitivity (photo-to-dark current ratio) is approximately 4.5 × 10^3^. After RTA at 700°C, the PC at 369 nm reaches a maximum value of 82.1 μA from that of 9.6 μA for the as-grown NWs and FWHM is reduced. As a consequence, the photosensitivity is increased to approximately 24.2 × 10^3^, leading to an enhancement of factor of five. With further RTA at 800°C, a similar PC is obtained (approximately 84.1 μA) with photosensitivity of approximately 24.2 × 10^3^. Note that the enhancement in photosensitivity is significant in this case and is similar to the earlier reports [4,5,10,13,14,16,27-29], as shown in Table 1 where more complex structures were fabricated by surface passivation of the ZnO NWs with different inorganic/organic materials. In our case, observed enhancement factor is quite high compared to the previously reported value for the conventional furnace annealed ZnO NWs [27]. Here, we obtained high photosensitivity by employing a simple RTA process, which shows the effectiveness of this process. In the PL spectra, we observed a reduction in oxygen-related defect states after RTA treatment. Therefore, RTA induced reduction in defect density is primarily responsible for the enhanced photoconduction, because very few photogenerated carriers can be trapped inside the defect states and most of the excess carriers contribute to the photocurrent. The responsivity of the as-grown ZnO NWs at wavelength 369 nm is calculated to be 6.8 for the as-grown NWs. After RTA, a major improvement in responsitivity by a factor of nine is observed. The inset of Figure 4c shows the UV light (wavelength 369 nm) intensity dependence of photosensitivity of the ZnO NWs, RTA treated at 800°C. The photosensitivity plot shows a sub-linear behavior with the UV light intensity. The observed sub-linear behavior is because of the complex interplay of electron-hole generation, carrier trapping, and recombination within the semiconductor NWS.

**Table 1:** The comparison of performances of the ZnO nanostructures-based photodetectors

Morphology	Device type	Light of detection (nm)	Bias (V)	Maximum photosensitivity	Photosensitivity enhancement factor from unmodified photodetector	Reference
Nanowires	Resistor	369	2.5	24200	Approximately 5.4	Present work
Nanowire	Resistor	365	5	10^4 ^- 10^6^	-	[4]
Thinfilm		365	5	31300	Approximately 4.2	[5]
Nanowires film	Resistor	254	5	17.7	-	[10]
Nanowires	Resistor	370	3	3367	Approximately 5.2	[13]
Nanowires film	Resistor	365	8	-	Approximately 4.7	[14]
Nanorod	Resistor	370	20	-	Approximately 3.0	[16]
Nanowires	Resistor	360	3	10^4^	Approximately 2.8	[27]
Nanowires	*n-i-n *junction	365	-5	1345	-	[28]
Nanowire	Resistor	390	5	10^4^	-	[29]

**Figure 5:**
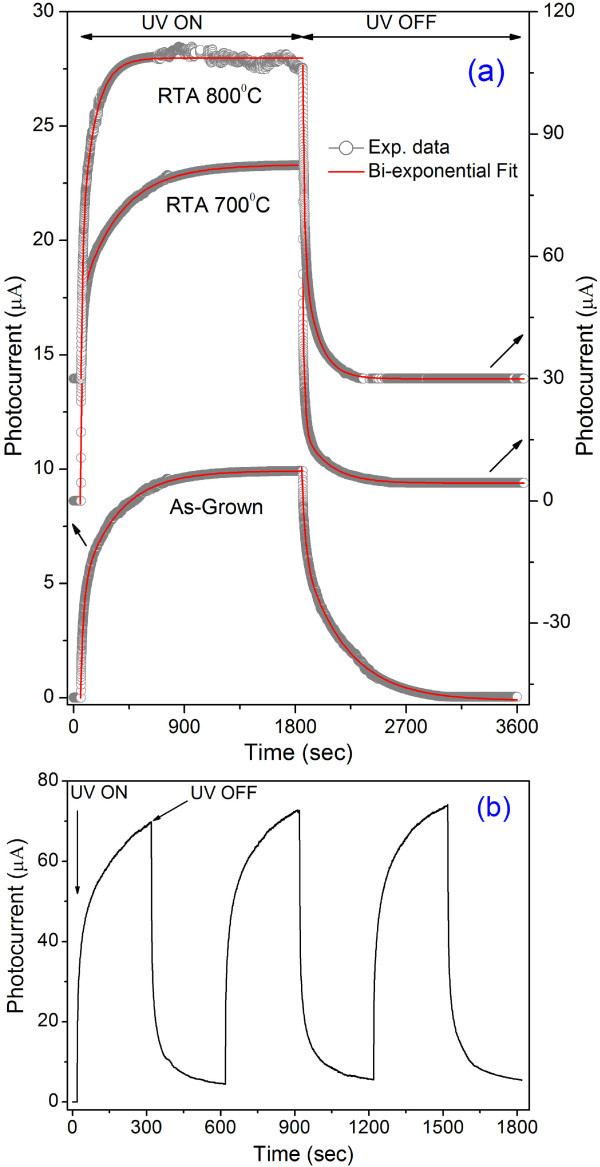
**The photocurrent growth and decay behaviors**. **(a) **The Photocurrent growth and decay behaviors of as-grown and RTA-treated ZnO NWs at a bias voltage of 2.5 V. Photocurrent growth and decay (photoresponse) are measured under the illumination of 360-nm UV light. **(b) **Photocurrent growth and decay of the RTA-treated ZnO NWs (700°C) under the pulsed (300 s) UV-light illumination at a bias voltage of 2.5 V.

To study further the effect of RTA on the photocurrent time response (growth and decay), we performed the photoresponse measurement of the as-grown and RTA-treated NWs under UV light (wavelength 360 nm) pulse in ON and OFF conditions and the results are shown in Figure 5. The photocurrent initially grows very fast and then slowly increased with time and saturated. The photoresponse of the ZnO NWs shows a bi-exponential growth and bi-exponential decay behavior. The time-dependent growth behavior of the photoresponse curve is fitted with the equation,

(1) $$I_{\text{ph}}\left(t\right)=I_{1}+A_{1}\left(1-e^{-t∕\tau_{1}}\right)-A_{2}e^{-t∕\tau_{2}}$$

where *I*_1_, *A*_1_, and *A*_2 _are positive constants. Calculated time constants from fittings are *τ*_1 _= 24.7 s and *τ*_2 _= 295.0 s for the as-grown NWs indicating a very rapid photocurrent growth initially followed by a very slow decay process. For the RTA-treated NWs, the photocurrent growth and decay time constants are 11.4 and 261.8 s for 700°C, and 12.3 and 107.0 s for 800°C. The photoresponse of ZnO consists of two parts: a rapid process of photogeneration and recombination of electron-hole pairs, and a slow process attributed to the surface adsorption and photodesorption of oxygen molecules [10,11]. In this process, the electron-hole generation is the only source for current carriers and all other processes decreased the carriers, this is explained in details later. Similarly, when the UV light is turned off, the photocurrent show a rapid decay followed by a slow decay. The time-dependent decay behavior can be fitted with a bi-exponential decay equation,

(2) $$I_{\text{ph}}\left(t\right)=I_{\text{ph}}\left(\infty \right)+A_{3}e^{-t∕\tau_{1}}+A_{4}e^{-t∕\tau_{2}}$$

where *A*_3 _and *A*_4 _are positive constants and *I*_ph_(∞) refers to the photocurrent after infinitely long time of the decay experiment, which essentially is the dark current. The decay time constants are calculated to be 25.7 and 347.9 s for the as-grown NWs, 12.3 and 298.5 s for the RTA treated at 700°C, 13.6 and 118.4 s for the RTA treated at 800°C, respectively. Therefore, the photocurrent growth as well as decay becomes faster after the RTA treatment. The calculations of individual time constants show that electron-hole recombination as well as generation rates become double after RTA at 700°C and do not change significantly for further annealing. On the other hand, oxygen adsorption rates during the photocurrent growth as well as decay are systematically decreases. Similar bi-exponential decay behavior with time constants of several seconds has been reported by several groups for the ZnO nanobelts, NWs, and thin film [1,5,11,14,30]. Figure 5b shows the photoresponse of the RTA-treated NWs under periodic UV illumination. The maximum photocurrent in the next cycle is slightly increased compared to the previous cycle because of the incomplete growth and decay of the PC during the measurement cycle. Second and third cycles of photocurrent growth and decay show exactly the replica of first cycle, indicating a PC response of the RTA-treated ZnO NWs, which is important for the real-time application in photodetctors.

**Figure 6:**
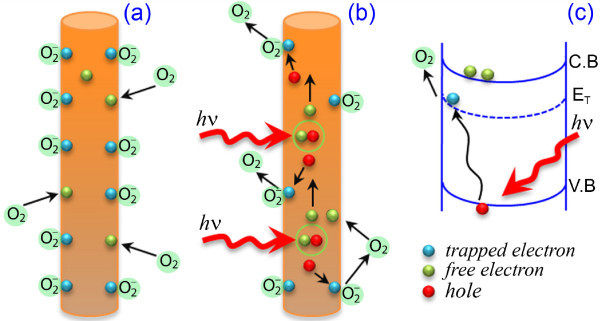
**A schematic of photoresponse mechanism of ZnO NWs**. **(a) **at dark condition and **(b) **during UV illumination. **(c) **Schematic energy band diagram of photoresponse process during UV illumination. The band bending is due to the formation of potential barrier in the NWs surface due to oxygen adsorption.

Bera et al. [27] and Ahn et al. [31] reported the degradation of photocurrent under steady UV illumination and humid air from the ZnO NWs. It was explained on the basis of the exchange of hydroxyl groups from absorbed water molecules and ionized oxygen at the surface defect sites. In the present case, we did not observed any degradation in the photocurrent under steady UV illumination, which eliminates the influence of water molecules on the photoresponse. As the NWs were grown at relatively higher temperature, there is a remote possibility of the presence of hydroxyl groups on the surface of the NWs. As the PL spectra indicate the presence of oxygen vacancy states on the surface, therefore oxygen is believed to play a critical role on the observed photoresponse. In normal condition, i.e., under the dark, the oxygen molecules from the air are easily stuck on the surface of the NWs by adsorption process due to the presence of trap levels and oxygen vacancy states on the surface. These chemisorbed oxygen molecules trapped the free electrons [O_2_(*g) *+ *e*^- ^*→ *O_2_^- ^] available on the surface near the Zn lattice and decreased the conductivity^4^, which is shown schematically in Figure 6a. This process leads to the formation of depletion layer near the surface resulting in the bending of conduction band and valence band, as well as trap-center-related band (E_T_) on upward direction (Figure 6c). Formation of large number of ionized oxygen on the NWs surface enhanced the band bending, resulting in a very low conductivity. During the UV illumination, electron-hole pairs are generated [hν *→ e^- ^*+ *h^+^*] by light absorption. Now these electrons/holes easily cross the depletion layers and contribute to the photoconduction process. At the same time, holes take part in the oxidization of ionized oxygen [O_2_^- ^+ *h*^+ ^*→ *O_2_(*g)*, photodesorption process] and release one oxygen gas molecule by electron-hole recombination process as shown schematically in Figure 6b. Then few of the released oxygen molecules re-adsorbed on the surface and decreased the free electron carriers. It is well known that the electron-hole generation is a rapid process, which results in a high PC in very short time; then it slowly reached the steady-state PC value by re-adsorption process. As the adsorption and photodesorption processes are slow processes [23], the PC reaches the saturation value very slowly. It is also know that adsorption process is slower than the photodesorption process. Therefore, during UV illumination, not all the holes are recombine with the electrons present in the ionized oxygen. As a result, holes are available for recombination with the exciton-related free electrons. During photocurrent decay, the exciton-related electron-hole recombination dominates, which corresponds to the faster decay component in the photocurrent, so the photocurrent initially decreases very rapidly. With the surface re-adsorption of oxygen due to the presence of free electron on the surface, the photocurrent comes to the initial value very slowly. As the RTA processing significantly reduces the surface defect-related trap centers and modified the surface of the ZnO NWs, the band bending is less here compared to the as-grown NWs case resulting in a comparatively higher conductivity, as revealed in the dark I-V characteristics. As a consequence, the photocurrent reached the saturation value very fast. Therefore, the defect/trap centers are responsible for the observed faster photocurrent growth and decay from the RTA-treated ZnO NWs. This is consistent with the PL results discussed earlier.

## Conclusion

We have shown a fivefold enhancement of photosensitivity in the UV region and faster photoresponse in RTA treated vertically aligned ZnO NWs, as compared to the as-grown NWs case. The photocurrent growth and decay rates (photoresponse) from RTA-treated NWs are improved by a factor of approximately 2. The dark current-voltage characteristics clearly indicate the presence of surface defects-related trap centers on the as-grown ZnO NWs and after RTA treatment it is significantly reduced. The RTA processing substantially removes the surface defect-related trap centers and modified the surface of the ZnO NWs, resulting in enhanced PC and faster photoresponse. The obtained results demonstrated that the RTA processing is an effective and simple way to achieve higher photosensitivity and relatively fast photoresponse, which is significant for the fabrication of ZnO NW based UV photodetectors.

## Abbreviations

RTA: rapid thermal annealing; NWs: nanowires; PC: photocurrent; PL: photoluminescence; FESEM: field emission scanning electron microscopy; HRTEM: high-resolution transmission electron microscopy.

## Competing interests

The authors declare that they have no competing interests.

## Authors' contributions

SD carried out all the experiments and analyses of the data. SD and PKG together interpreted the results and prepared the manuscript.

## Supplementary Material

Additional file 1 **Analysis of photoluminescence data of ZnO NWs**. Supplementary information of detailed analysis of photoluminescence data of ZnO NWs. http://www.nanoscalereslett.com/imedia/1374497915579958/supp1.doc Click here for file
