# Supplementary material for: Enhanced UV photosensitivity from rapid thermal annealed vertically aligned ZnO nanowires
Source: Nanoscale Res Lett. 2011 Aug 22;6(1):504. doi: 10.1186/1556-276X-6-504 (PMC3212019; doi:10.1186/1556-276X-6-504)
Supplement: Additional file 1 — Analysis of photoluminescence data of ZnO NWs. Supplementary information of detailed analysis of photoluminescence data of ZnO NWs. http://www.nanoscalereslett.com/imedia/1374497915579958/supp1.doc [file 1556-276X-6-504-S1.DOC]

**Supplementary Information**

**Enhanced UV Photosensitivity from Rapid Thermal Annealed Vertically aligned ZnO Nanowires**

*Soumen Dhara and P. K. Giri*

**Analysis of Photoluminescence Data of ZnO NWs:**

**Figure S**: Room temperature PL spectra of the: (a) as-grown; (b) RTA treated at 700◦C; and (c) 800°C ZnO NWs. Solid red lines are the fitted peaks with Gaussian profile to the experimental data and blue lines are the corresponding individual peak components. Fitted peak positions are mentioned on the graph in nm unit.

**Table S**: Summary of the fitted peak parameters for PL spectra of the as-grown and RTA treated ZnO NWs

| **ZnO NWs** | **PL Peak positions (Amplitude)** | | | |
| --- | --- | --- | --- | --- |
| As-grown | 380 (6.03) | - | 500 (3.02) | 545 (3.39) |
| RTA 700°C | 379 (17.1) | 394 (3.93) | 500 (3.54) | - |
| RTA 800°C | 379 (17.97) | 394 (4.37) | 500 (1.84) | - |
